# Concordance of PD‐1 and PD‐L1 (B7‐H1) in paired primary and metastatic clear cell renal cell carcinoma

**DOI:** 10.1002/cam4.2769

**Published:** 2019-12-12

**Authors:** Jeanette E. Eckel‐Passow, Thai H. Ho, Daniel J. Serie, John C. Cheville, R. Houston Thompson, Brian A. Costello, Haidong Dong, Eugene D. Kwon, Bradley C. Leibovich, Alexander S. Parker

**Affiliations:** ^1^ Division of Biomedical Statistics and Informatics Mayo Clinic Rochester MN USA; ^2^ Division of Hematology and Medical Oncology Mayo Clinic Scottsdale AZ USA; ^3^ Department of Health Sciences Research Mayo Clinic Jacksonville FL USA; ^4^ Laboratory Medicine and Pathology Mayo Clinic Rochester MN USA; ^5^ Department of Urology Mayo Clinic Rochester MN USA

**Keywords:** B7-H1, ccRCC, PD-1, PD-L1, RCC

## Abstract

**Objectives:**

Previous studies noted discordance of programmed death‐1 (PD‐1) and one of its ligands (PD‐L1) across patient‐matched primary and metastatic clear cell renal cell carcinoma (ccRCC). There are inconsistencies if the primary or metastatic tumor has higher expression, and whether metastatic tumor expression is associated with patient outcome. Thus, we examined PD‐1 and PD‐L1 in patient‐matched tumors using a large number of ccRCC patients with long follow‐up.

**Materials and Methods:**

We analyzed PD‐1 and PD‐L1 using immunohistochemistry in patient‐matched primary and metastatic tumors from 110 ccRCC patients. Concordance was assessed among longitudinal metastatic tumors, as well as across patient‐matched primary and metastatic tumors. Cox proportional hazards regression was used to evaluate the associations of metastatic tumor expression with cancer‐specific survival.

**Results:**

We observed inter‐metastatic tumor heterogeneity of PD‐1 in 25 (69%) of the 36 patients and of PD‐L1 in seven (19%) patients. Concordance between patient‐matched primary and metastatic tumors was 73% (Kappa = 0.16, 95% CI: −0.003‐0.32). Similarly, concordance of PD‐L1 between metastatic and patient‐matched primary tumors was 78% (Kappa = 0.27, 95% CI: 0.09‐0.46). Both markers demonstrated higher expression in primary vs metastatic tumors. Metastatic tumor expression of PD‐1 was significantly associated with metastatic location (*P* < .0001) and ccRCC‐specific survival (HR = 2.15, 95% CI: 1.06‐4.36, *P* = .035).

**Conclusions:**

The expression of PD‐1 and PD‐L1 is discordant across patient‐matched ccRCC tumors, with higher expression in primary tumors. Higher PD‐1 expression was associated with metastatic location and lower cancer‐specific survival. If validated, these results highlight the importance of evaluating these biomarkers in metastatic tissue specifically.

## Graphical abstract

We examined PD‐1 and PD‐L1 in patient‐matched tumors in a large cohort of ccRCC with long‐term follow‐up. The expression of PD‐1 and PD‐L1 was discordant across patient‐matched ccRCC tumors, with higher expression in primary tumors. Higher PD‐1 expression was associated with metastatic location and lower cancer‐specific survival.

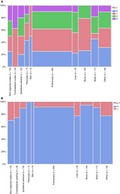

## 1 INTRODUCTION

Metastatic clear cell renal cell carcinoma (ccRCC) standard of care is quickly evolving to include immune checkpoint inhibitors, which target programmed death 1 (PD‐1).^1^, ^2^ While immune checkpoint inhibitors have been successful in advanced ccRCC patients, not all patients respond to these inhibitors. Thus, there is an important clinical need to identify biomarkers for these promising therapies.^3^, ^4^ Related to this, while higher PD‐1 and PD‐L1 expressions in primary tumors have shown to predict poor survival,^5^, ^6^, ^7^ their role in selecting patients for immune checkpoint inhibitors remains unclear.^8^, ^9^, ^10^ Of particular interest, many studies evaluating these biomarkers have focused on the expression in the primary ccRCC tumor, vs the metastatic tumor that is more therapeutically relevant. Indeed, this has raised recent interest in the important questions of whether PD‐1 and PD‐L1 expressions are similar in primary and metastatic ccRCC from the same patient, and whether expression in the metastatic tumor is associated with survival.

Motivated by these questions, Jilaveanu et al^11^ compared PD‐L1 expression using tissue microarrays on 34 ccRCC patient pairs and observed weak correlation across primary and metastatic tumors. Similarly, Callea et al^12^ observed discordant PD‐L1 expression (via immunohistochemistry [IHC]) in 21% of 53 ccRCC pairs. More recently, Zhang et al^13^ compared PD‐1 and PD‐L1 expressions (via IHC) in patient‐matched tumors from 165 Asian RCC patients (78% ccRCC) and observed discordant PD‐1 and PD‐L1 across primary and metastatic tumors in lung/lymph node metastases, PD‐L1 in bone metastases, and PD‐1 in brain and viscera metastases. Interestingly, Zhang and colleagues were the first to report a significant association of PD‐L1 metastatic tumor expression with overall survival; however, they did not observe a significant association with PD‐1 and overall survival. While all three studies evaluated patient‐matched tumors, Callea^12^ observed higher expression in primary tumors, whereas Jilaveanu^11^ and Zhang^13^ observed higher expression in metastatic tumors. Given the inconsistencies reported to date, we evaluated a large cohort of ccRCC patients to confirm that the expression of PD‐1 and PD‐L1 is discordant across patient‐matched primary and metastatic ccRCC tumors, with higher expression in the primary tumors. More importantly, we are the first to report that higher expression of PD‐1 in metastatic ccRCC is associated with timing of metastasis and poorer cancer‐specific survival.

## 2 MATERIALS AND METHODS

### 2.1 Patient selection and pathology review

We identified 110 patients who had a nephrectomy between 1990 and 2005 at Mayo Clinic Rochester, had a metastasectomy for at least one metastatic tumor and formalin‐fixed, paraffin‐embedded (FFPE) tissue was available from their primary tumor, and at least one metastatic tumor. Contralateral renal tumors and multifocal renal tumors were not considered as metastatic. All tumors were comprehensively reviewed by one pathologist (JCC) to confirm 2016 WHO histological subtype, 2016 WHO/ISUP grade, 2017 AJCC TNM prognostic stage groups, tumor size, and the presence of sarcomatoid differentiation and coagulative tumor necrosis. All FFPE block(s) that were representative of the tumor (highest grade) were utilized; the block with the highest PD‐1 or PD‐L1 score was retained for analysis. The Mayo Clinic IRB approved this study.

### 2.2 PD‐1 and PD‐L1

**Figure 1:**
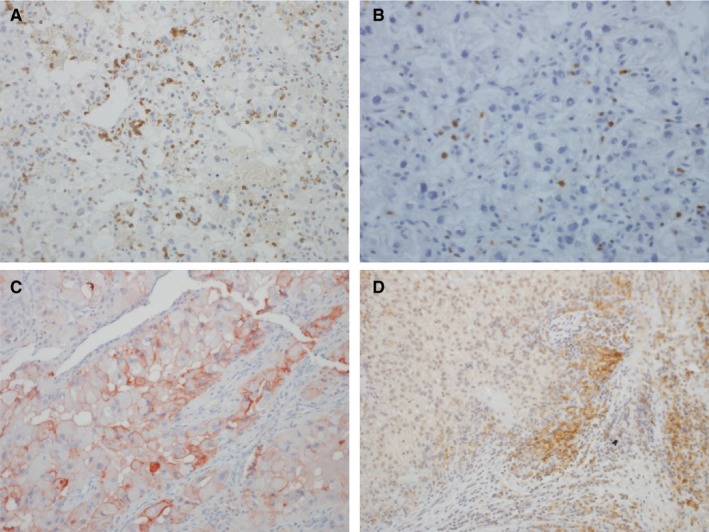
Immunohistochemistry illustration for (A) PD‐1 in primary ccRCC, small lymphocytes are PD‐1‐positive; (B) PD‐1 in metastatic ccRCC; (C) PD‐L1 in primary ccRCC with membrane staining of the neoplastic cells; and (D) PD‐L1 in metastatic ccRCC highlighting staining of the mononuclear inflammatory cells

Five‐micrometer thick FFPE sections were stained for loss of expression of PD‐1 and PD‐L1. Briefly, after deparaffinization of FFPE slides and blocking of endogenous peroxidase, slides were incubated with anti‐human PD‐1 antibody (R&D Systems, goat polyclonal antibody) at a dilution of 1:100 for primary tumors and mouse anti‐human‐PD‐1 antibody (Abcam) for metastatic tumors at a dilution of 1:500, both in Da Vinci Green antibody diluent (Biocare Medical) for 30 minutes at room temperature. Mouse anti‐human PD‐L1 from Dong Lab was used for both primary and metastatic tumors with a dilution at 1:100‐1:300 for 60 minutes at room temperature. Stained slides were reviewed by a single pathologist in a blinded fashion (Figure 1). In each case, there was an internal positive control (PD‐1‐positive lymphocytes, and PD‐L1‐positive lymphocytes or macrophages). For PD‐1, tumor‐infiltrating mononuclear immune cells were considered positive if there was histologic evidence of cell surface membrane staining.^6^ For PD‐L1, the tumor cells, or tumor‐infiltrating mononuclear immune cells, were considered positive if there was histologic evidence of cell surface membrane staining. PD‐1 was quantified as absent, focal, moderate, and marked. PD‐L1 was quantified as percentage of cells staining in 5%‐10% increments. For analyses, PD‐L1 was dichotomized as absent (0% cell staining) and present (>0% cells staining).

### 2.3 Statistical methods

PD‐1 and PD‐L1 protein expressions were summarized across primary and metastatic tumors. Fisher's exact test was used to test for an association between metastatic expression of PD‐1 and PD‐L1 with metastatic location. Cohen's Kappa was used to measure the agreement between patient‐matched primary and metastatic tumors; Kappa accounts for agreement arising by chance. Kappa value less than zero indicates no agreement, 0‐0.20 slight, 0.21‐0.40 fair, 0.41‐0.60 moderate, 0.61‐0.80 substantial, and 0.81‐1 nearly perfect agreement. To determine if the expression in metastatic tumors was associated with ccRCC‐specific survival, Cox proportional hazards regression was used. Cox models were adjusted for age at metastatic diagnosis, and dichotomized IHC staining was included as a time‐dependent covariate. *P*‐values < .05 were deemed statistically significant.

## 3 RESULTS

### 3.1 Patient characteristics

**Figure 2:**
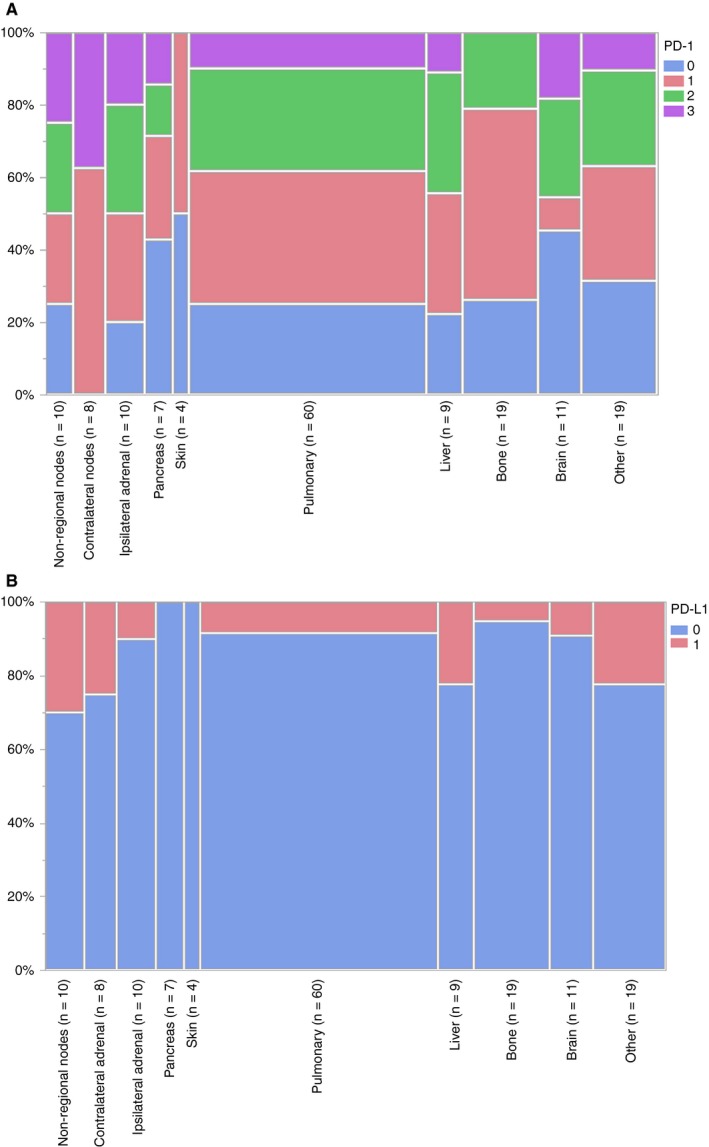
Metastatic expression of (A) PD‐1 by metastatic location (Fisher's exact test *P* < .0001) and (B) PD‐L1 by metastatic location (Fisher's exact test *P* = .29)

The cohort included 110 ccRCC patients with a primary tumor and at least one metastatic tumor available for analysis (Table 1). PD‐1 staining was successful for 105 primary ccRCC tumors; 11% did not express PD‐1. PD‐L1 staining was successful for 97 primary ccRCC tumors; 75% did not express PD‐L1. Of the 110 patients, 56 (51%) had synchronous (M0) and 54 (49%) metachronous (M1) ccRCC metastases. Median time from nephrectomy to first metachronous metastasis was 1.7 years (min = 31 days, max = 10.7 years). From the 110 patients, 157 patient‐matched metastases were analyzed: 74 patients had one, 27 patients had two, seven patients had three, and two patients had four metastatic tumors analyzed (Table 1). Pulmonary (38%) and bone (12%) were the most common metastatic locations (Table 2). Metastatic expression of PD‐1 was significantly associated with metastatic location (*P* < .0001, Figure 2A); however, we did not observe a significant association of PD‐L1 with metastatic location (*P* = .29, Figure 2B).

**Table 1:** Clinical and pathology characteristics associated with the primary ccRCC tumor

	M0 at presentation (N = 56)	M1 (N = 54)	Total (N = 110)
Gender			
Female	15 (26.8%)	14 (25.9%)	29 (26.4%)
Male	41 (73.2%)	40 (74.1%)	81 (73.6%)
Age at surgery (y)			
Mean	62.1	58.5	60.3
Median	64.2	59.1	61.5
Range	(34.9‐78.8)	(38.2‐73.7)	(34.9‐78.8)
Max tumor size (cm)			
Mean	9.4	10.9	10.1
Median	9.0	10.0	9.5
Range	(2.5‐18.0)	(2.1‐23.0)	(2.1‐23.0)
2017 AJCC TNM prognostic stage groups			
I	11 (19.6%)	0 (0.0%)	11 (10.0%)
II	18 (32.1%)	0 (0.0%)	18 (16.4%)
III	25 (44.6%)	0 (0.0%)	25 (22.7%)
IV	2 (3.6%)	54 (100.0%)	56 (50.9%)
Grade			
1	1 (1.8%)	1 (1.9%)	2 (1.8%)
2	14 (25.0%)	5 (9.3%)	19 (17.3%)
3	31 (55.4%)	31 (57.4%)	62 (56.4%)
4	10 (17.9%)	17 (31.5%)	27 (24.5%)
Necrosis			
No	29 (51.8%)	16 (29.6%)	45 (40.9%)
Yes	27 (48.2%)	38 (70.4%)	65 (59.1%)
SSIGN score			
Missing	0	1	1
0‐3	11 (19.6%)	0 (0.0%)	11 (10.1%)
4‐7	38 (67.9%)	7 (13.2%)	45 (41.3%)
8+	7 (12.5%)	46 (86.8%)	53 (48.6%)
PD‐1 IHC in primary tumor			
Missing	3	2	5
Absent	6 (11.3%)	6 (11.5%)	12 (11.4%)
Focal	26 (49.1%)	24 (46.2%)	50 (47.6%)
Moderate	13 (24.5%)	16 (30.8%)	29 (27.6%)
Marked	8 (15.1%)	6 (11.5%)	14 (13.3%)
PD‐L1 IHC in primary tumor (Continuous)			
Missing	10	3	13
Mean	1.8	8.3	5.1
Median	0	0	0
Range	(0,30)	(0,100)	(0,100)
PD‐L1 IHC in primary tumor (dichotomized)			
Missing	10	3	13
Absent (=0)	37 (80.4%)	36 (70.6%)	73 (75.3%)
Present (>0)	9 (19.6%)	15 (29.4%)	24 (24.7%)
Number of metastases			
1	33 (58.9%)	41 (75.9%)	74 (67.3%)
2	15 (26.8%)	12 (22.2%)	27 (24.5%)
3	6 (10.7%)	1 (1.9%)	7 (6.4%)
4	2 (3.6%)	0 (0.0%)	2 (1.8%)

**Table 2:** Clinical and pathology characteristics associated with the metastatic tumors

	M0 (N = 89)	M1 (N = 68)	Total (N = 157)
Metastatic site			
Contralateral adrenal	3 (3.4%)	5 (7.4%)	8 (5.1%)
Ipsilateral adrenal	2 (2.2%)	8 (11.8%)	10 (6.4%)
Pancreas	5 (5.6%)	2 (2.9%)	7 (4.5%)
Skin	2 (2.2%)	2 (2.9%)	4 (2.5%)
Non‐regional nodes	9 (10.1%)	1 (1.5%)	10 (6.4%)
Pulmonary	40 (44.9%)	20 (29.4%)	60 (38.2%)
Liver	4 (4.5%)	5 (7.4%)	9 (5.7%)
Bone	9 (10.1%)	10 (14.7%)	19 (12.1%)
Brain	7 (7.9%)	4 (5.9%)	11 (7.0%)
Other*	8 (9.0%)	11 (16.2%)	19 (12.1%)
Metastatic grade			
2	16 (18.0%)	13 (19.1%)	29 (18.5%)
3	59 (66.3%)	36 (52.9%)	95 (60.5%)
4	14 (15.7%)	19 (27.9%)	33 (21.0%)
Metastatic necrosis			
No	56 (62.9%)	38 (55.9%)	94 (59.9%)
Yes	33 (37.1%)	30 (44.1%)	63 (40.1%)
Metastatic sarcomatoid			
No	85 (95.5%)	62 (91.2%)	147 (93.6%)
Yes	4 (4.5%)	6 (8.8%)	10 (6.4%)
PD‐1 IHC in metastatic tumor			
Missing	2	0	2
Absent	23 (26.4%)	19 (27.9%)	42 (27.1%)
Focal	29 (33.3%)	27 (39.7%)	56 (36.1%)
Moderate	21 (24.1%)	17 (25.0%)	38 (24.5%)
Marked	14 (16.1%)	5 (7.4%)	19 (12.3%)
PD‐L1 IHC in metastatic tumor			
Missing	0	1	1
Absent	77 (86.5%)	60 (89.6%)	137 (87.8%)
Present	12 (13.5%)	7 (10.4%)	19 (12.2%)

* Other includes thyroid, bowel, spleen, muscle, omentum, heart, etc

### 3.2 Concordance of longitudinal metastatic ccRCC tumors

**Figure 3:**
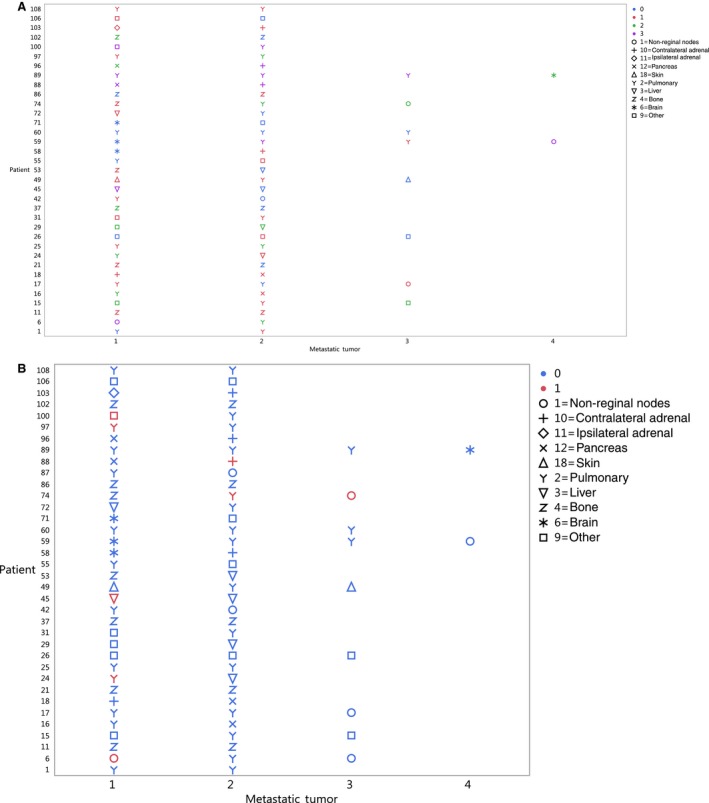
(A) PD‐1 IHC staining was performed on longitudinal metastatic tumors from 36 patients. PD‐1 staining is denoted as blue 0 = absent, red 1 = focal, green 2 = moderate, and purple 3 = marked. (B) PD‐L1 staining was performed on longitudinal metastatic tumors from 36 patients. Blue denotes PD‐L1 staining is absent and red denotes PD‐L1 staining is present. Symbols denote the metastatic location

PD‐1 and PD‐L1 were quantified on longitudinal metastatic tumors from 36 patients. Inter‐metastatic tumor heterogeneity of PD‐1 was observed in 25 (69%) of the 36 patients (Figure 3A) and of PD‐L1 in seven (19%) patients (Figure 3B).

### 3.3 Concordance of patient‐matched primary and metastatic ccRCC tumors

#### 3.3.1 PD‐1

A total of 146 metastatic tumors and 105 patient‐matched primary tumors were stained for PD‐1. Overall concordance between the 146 metastatic tumors and the patient‐matched primary tumors was 34% (Kappa = 0.077, 95% CI: −0.032‐0.19): 32% in metachronous and 36% in synchronous metastatic tumors (Table 3). If PD‐1 was dichotomized as absent vs present, the concordance was 73% (Kappa = 0.16, 95% CI: −0.003‐0.32): 72% in metachronous and 75% in synchronous metastatic tumors.

**Table 3:** Concordance of PD‐1 staining between patient‐matched primary and metastatic tumors

Primary tumor	Metastatic tumor			
	Absent	Focal	Moderate	Marked
Absent	8	7	1	0
Focal	26	21	17	4
Moderate	3	19	11	6
Marked	2	4	8	9

#### 3.3.2 PD‐L1

PD‐L1 staining was successful for 140 metastatic tumors and 97 patient‐matched primary tumors. Overall concordance between the 140 metastatic tumors and the patient‐matched primary tumors was 78% (Kappa = 0.27, 95% CI: 0.09‐0.46): 78% in synchronous and 78% in metachronous metastatic tumors (Table 4). We observed a significant association of the difference in expression between patient‐matched primary‐metastatic tumor pairs and metastatic tumor timing, with synchronous metastatic tumors being more different than their matched primary tumor than metachronous metastatic tumors (*P* = .04; Table S1).

**Table 4:** Concordance of PD‐L1 staining between patient‐matched primary and metastatic tumors

Primary Tumor	Metastatic Tumor	
	Absent	Present
Absent	99	8
Present	23	10

### 3.4 Co‐expression of PD‐1 and PD‐L1

There was a significant association between the expression of PD‐1 and PD‐L1 in the primary tumors (*P* = .042): eight (9%) did not express either PD‐1 or PD‐L1, 63 (68%) only expressed PD‐1, zero only expressed PD‐L1, and 22 (24%) expressed both PD‐1 and PD‐L1 (*P* = .042; Table S2). Similarly, there was a significant association between the expression of PD‐1 and PD‐L1 in the metastatic tumors (*P* < .0001): 41 (27%) did not express either PD‐1 or PD‐L1, 94 (61%) only expressed PD‐1, one (<1%) only expressed PD‐L1, and 18 (12%) expressed both PD‐1 and PD‐L1 (*P* < .0001; Table S3).

### 3.5 Metastatic tumor expression and RCC‐specific survival

PD‐1 metastatic tumor expression was significantly associated with ccRCC‐specific survival (HR = 2.15, 95% CI: 1.06‐4.36, *P* = .035) after adjustment for age at metastatic diagnosis. We did not observe a statistically significant association between PD‐L1metastatic tumor expression and ccRCC‐specific outcome (HR = 1.37, 95% CI: 0.75‐2.53, *P* = .31).

## 4 DISCUSSION

As the use of immune checkpoint inhibitors in ccRCC increases, there is a need to better understand whether tumor‐based PD‐1 and PD‐L1 expressions represent logical biomarkers to guide use of these therapies. Stifling this effort are conflicting data regarding concordance between PD‐1 and PD‐L1 expressions in primary and metastatic tumors from the same patient, and lack of data on whether expression of these markers in metastatic ccRCC is associated with cancer‐specific survival. We advance the field by confirming that PD‐1 and PD‐L1 are discordant across patient‐matched primary and metastatic ccRCC tumors, and provide further evidence that the expression of both markers is higher in primary tumors. We also report for the first time that the expression of PD‐1 in metastatic ccRCC tumors is associated with poor cancer‐specific survival.

With respect to discordance of patient‐matched primary and metastatic ccRCC tumors, 11% of the primary tumors in our cohort did not express PD‐1, and 75% did not express PD‐L1. In contrast, 27% of the metastatic tumors did not express PD‐1, and 88% did not express PD‐L1. Other investigators have similarly observed discordance of PD‐1 and PD‐L1 across patient‐matched primary and metastatic RCC tumors^11^, ^12^, ^13^; however, with different directions. Similar to our observations, Callea et al^12^ observed higher expression (via IHC) in primary tumors, whereas Jilaveanu et al^11^ (via tissue microarray) and Zhang et al^13^ (via IHC) observed higher expression in metastatic tumors. One possible explanation for the difference in direction is that while the patients described herein as well as those by Callea^12^ were all ccRCC, the patients analyzed by Zhang^13^ and Jilaveanu^11^ included both ccRCC and non‐ccRCC. Of note, we also observed that the expression difference across primary and metastatic tumors for PD‐L1 was associated with metastatic tumor timing; synchronous metastatic patients had larger differences between their primary and metastatic tumor pairs in comparison to metachronous metastatic patients. Thus, distant metachronous metastases that develop after resection of the primary tumor may have evolved independently of the primary tumor. Taken in consort, this confirmation of discordance and higher expression in primary ccRCC is important given that it could provide a broad explanation for why these biomarkers are not currently viewed as valuable in guiding the use of immune checkpoint inhibitors. That is, if the expression of either marker in primary tumors is being used as a guide for response to immune checkpoint inhibitors, we confirm that this would provide misleading information on what is being expressed in the metastatic tumor (ie, the actual target of these therapies). Moreover, our validation that expression is higher in primary tumors suggests that the direction of this misinformation would be to label patients as potential responders who are actually unlikely to respond (ie, their primary tumor expresses therapeutically relevant levels of the target, but their metastatic tumor does not). In CHECKMATE 214, a randomized study between combination nivolumab/ipilimumab vs sunitinib, positive PD‐L1 expression (defined as ≥ 1%), was not predictive of favorable responses to immunotherapy.^2^ In fact, 7% of patients with PD‐L1‐negative tumors had a complete response to combination immunotherapy compared to 1% for the antiangiogenic therapy control arm.

Another important consideration is concordance across longitudinal metastatic tumors. With respect to intra‐metastatic tumor heterogeneity of PD‐L1, Callea^12^ analyzed multiple metastatic tumors from 14 patients and observed that only one (7%) patient was discordant for PD‐L1. We analyzed multiple metastatic tumors from 36 patients and observed discordant PD‐L1 in 19%, and discordant PD‐1 in 69%, of the patients. Notably, the tissue block analyzed for each sample was identified by our pathologist as the most representative of the tumor, that is, highest grade. This aligns with recommendations based on observations that PD‐L1 expression is associated with high nuclear grade.^12^ These data underscore that when using PD‐L1 and PD‐1 expressions as guides for use of checkpoint inhibitors it is important to be mindful that in a patient with multiple metastases, even if one lesion expresses the target, this does not mean that all lesions will uniformly express the target.

Few investigators have evaluated whether PD‐L1 and PD‐1 expressions are associated with the location of the metastatic lesion. Zhang et al^13^ reported that metastatic expression of PD‐L1 is higher in pulmonary and lymph node, and lower in brain and visceral metastases. In contrast, we did not observe a significant association between PD‐L1 and metastatic location. However, we observed a significant association between the expression of PD‐1 and metastatic location, with PD‐1 expression being highest in contralateral nodes. Related to this, co‐expression of PD‐1 and PD‐L1 has been evaluated across a range of solid tumors, with a reported prevalence of 33% in primary kidney tumors.^14^ We observed similar prevalence of co‐expression of PD‐1 and PD‐L1 in primary ccRCC tumors: 24% expressed both PD‐1 and PD‐L1, and 9% did not express either PD‐1 or PD‐L1. In metastatic tumors, 12% expressed both PD‐1 and PD‐L1, and 27% did not express either PD‐1 or PD‐L1. Co‐expression of PD‐1 and PD‐L1 was observed at a prevalence of 50% in a subset of ccRCC tumors with sarcomatoid differentiation.^15^ Thus, for PD‐1‐based assays, there may be variations in the same patient with regard to PD‐1 expression relative to the metastatic site being biopsied.

PD‐1 and PD‐L1 expressions in primary ccRCC have been reported to be associated with poor patient outcome and pathologic features associated with aggressive tumors.^5^, ^6^, ^7^ From a therapeutic standpoint, expression of these biomarkers in the primary tumor does not represent the most relevant evaluation. Rather, the association between the expression in metastatic ccRCC and outcome is more therapeutically relevant. While Zhang et al^13^ reported that PD‐L1 metastatic expression was associated with poorer overall survival, the use of death from any cause is not the most robust endpoint for these analyses. We observed that PD‐1 metastatic tumor expression was significantly associated with poorer cancer‐specific survival; however, we did not observe a statistically significant association with PD‐L1 metastatic tumor expression and cancer‐specific survival. The differing results between these studies could reflect the different endpoints being evaluated: overall survival vs cancer‐specific survival.

There are some limitations associated with our study. Mainly, there are likely biological differences between patients who are eligible for metastasectomy vs patients ineligible for metastasectomy. Prior studies have demonstrated that ccRCC with pancreatic metastases has more of an indolent course when compared to ccRCC without pancreatic metastases. In contrast, patients with more aggressive disease or sarcomatoid differentiation are less likely to undergo metastasectomy. Thus, patients with more aggressive disease or ineligible for metastasectomy may not be accurately represented in our analyses. While we used the same monoclonal antibody for PD‐L1 in both primary and metastatic tumors, we used different antibodies for PD‐1 due to the availability of a commercial antibody to human PD‐1 at the time each cohort was analyzed. Specifically, we used a polyclonal antibody of PD‐1 for primary tumors and monoclonal antibody of PD‐1 for metastatic tumors.

In conclusion, we validated that the expression of PD‐1 and PD‐L1 was discordant across patient‐matched primary and metastatic ccRCC tumors, with higher expression being observed in primary ccRCC tumors. We also observed associations of PD‐1 expression with metastatic location and cancer‐specific survival.

## CONFLICT OF INTEREST

One or more of the investigators associated with this project and Mayo Clinic have a financial interest in technology used in the research and may stand to gain financially from the successful outcome of the research.

## Author Contributions

Jeanette Eckel‐Passow: Conceptualization, statistical analysis, writing. Thai Ho: Investigation, writing. Daniel Serie: Statistical analysis. John Cheville: Investigation, data curation. R. Houston Thompson: Investigation, data curation. Brian Costello: Investigation, data curation. Haidong Dong: Methodology, data curation, writing. Eugene Kwon: Conceptualization, investigation, data curation. Bradley Leibovich: Investigation, data curation, writing. Alexander S. Parker: Conceptualization, methodology, writing and critical revisions, supervision, and funding.

## Data Availability

We will make data available upon request.
