# Supplementary material for: Concordance of PD‐1 and PD‐L1 (B7‐H1) in paired primary and metastatic clear cell renal cell carcinoma
Source: Cancer Med. 2019 Dec 12;9(3):1152–60. doi: 10.1002/cam4.2769 (PMC6997072; doi:10.1002/cam4.2769)
Supplement: Supplementary file 1 [file CAM4-9-1152-s001.docx]

**SUPPLEMENTARY MATERIALS**

**PD-1 and PD-L1 (B7-H1) in Metastatic Clear Cell Renal Cell Carcinoma and Concordance with Paired Primary Tumor**

Jeanette E. Eckel-Passow, PhD^a^, Thai H. Ho, MD, PhD^b^, Daniel J. Serie, BS^c^, John C. Cheville, MD^d^, R. Houston Thompson, MD^e^, Brian A. Costello, MD^e^, Haidong Dong, MD, PhD^e^, Eugene D. Kwon, MD^e^, Bradley C. Leibovich, MD^e^, Alexander S. Parker, PhD^f#^

^a^Division of Biomedical Statistics and Informatics, Mayo Clinic, Rochester, MN

^b^Division of Hematology and Medical Oncology, Mayo Clinic, Scottsdale, AZ

^c^Department of Health Sciences Research, Mayo Clinic, Jacksonville, FL

^d^Laboratory Medicine and Pathology, Mayo Clinic, Rochester, MN

^e^Department of Urology, Mayo Clinic, Rochester, MN

^f^Division of Hematology/Oncology, Mayo Clinic, Jacksonville, FL

**Supplementary Table S1**: Association of the difference in IHC staining between metastatic-primary tumor pairs with metastatic tumor features for PD-1 and PD-L1. PD-1 and PD-L1 were dichotomized as absent versus present.

|  | **PD-1** | | | |  | **PD-L1** | | | |
| --- | --- | --- | --- | --- | --- | --- | --- | --- | --- |
|  | **Mean Difference** | **Estimate** | **Estimate Pvalue** | **Global F-Test Pvalue** |  | **Mean Difference** | **Estimate** | **Estimate Pvalue** | **Global F-Test Pvalue** |
| **Metastatic Tumor Site** |  |  |  | 0.114 |  |  |  |  | 0.233 |
| PULMONARY | -0.105 | Ref | -- |  |  | 4.180 | Ref | -- |  |
| BONE | -0.053 | 0.073 | 0.785 |  |  | -0.882 | -7.829 | 0.099 |  |
| BRAIN | -0.727 | -0.684 | 0.029 |  |  | 5.900 | 2.023 | 0.703 |  |
| CONTRALATERAL ADRENAL | 0.143 | 0.306 | 0.416 |  |  | 10.000 | 5.669 | 0.344 |  |
| IPSILATERAL ADRENAL | -0.100 | 0.055 | 0.868 |  |  | -5.500 | -9.531 | 0.089 |  |
| LIVER | -0.111 | -0.013 | 0.969 |  |  | 0.556 | -4.668 | 0.416 |  |
| NON-REGIONAL NODES | 0.125 | 0.188 | 0.575 |  |  | 8.000 | 3.875 | 0.460 |  |
| OTHER | -0.600 | -0.379 | 0.171 |  |  | -4.438 | -7.875 | 0.099 |  |
| PANCREAS | -0.333 | -0.261 | 0.516 |  |  | -0.833 | -7.143 | 0.290 |  |
| SKIN | -1.750 | -1.274 | 0.016 |  |  | 0.000 | -3.805 | 0.650 |  |
| **Metastatic Tumor Timing** |  |  |  | 0.518 |  |  |  |  | **0.040** |
| M0 | -0.171 | Ref | -- |  |  | 4.776 | Ref | -- |  |
| M1 | -0.297 | -0.116 | 0.518 |  |  | -1.188 | -5.878 | 0.040 |  |
| **Metastatic Tumor Grade** |  |  |  | 0.450 |  |  |  |  | 0.213 |
| 2 | -0.192 | Ref | -- |  |  | 0.893 | Ref | -- |  |
| 3 | -0.193 | -0.026 | 0.912 |  |  | 0.213 | -0.298 | 0.934 |  |
| 4 | -0.344 | -0.274 | 0.316 |  |  | 7.656 | 5.511 | 0.196 |  |
| **Metastatic Tumor Necrosis** |  |  |  | 0.318 |  |  |  |  | 0.064 |
| No | -0.261 | Ref | -- |  |  | 0.000 | Ref | -- |  |
| Yes | -0.172 | 0.169 | 0.318 |  |  | 5.315 | 5.233 | 0.064 |  |
| **Metastatic Tumor Sarcomatoid** |  |  |  | 0.356 |  |  |  |  | 0.101 |
| No | -0.206 | Ref | -- |  |  | 1.362 | Ref | -- |  |
| Yes | -0.500 | -0.306 | 0.356 |  |  | 11.000 | 8.765 | 0.101 |  |
|  |  |  |  |  |  |  |  |  |  |

**Supplementary Table S2.** Co-expression of PD-1 and PD-L1 across 93 primary tumors (Fisher’s exact test: p=0.042).

|  | | | | | | |
| --- | --- | --- | --- | --- | --- | --- |
| **PD-L1** | **PD-1** | | | | |  |
|  | **Absent** | **Focal** | **Moderate** | **Marked** |  |  |
| **Absent** | 8 | 39 | 17 | 7 |  |  |
| **Present** | 0 | 8 | 8 | 6 |  |  |
|  |  |  |  |  |  |  |

**Supplementary Table S3.** Co-expression of PD-1 and PD-L1 across 154 metastatic tumors (Fisher’s exact test: p<0.0001).

|  | | | | | | |
| --- | --- | --- | --- | --- | --- | --- |
| **PD-L1** | **PD-1** | | | | |  |
|  | **Absent** | **Focal** | **Moderate** | **Marked** |  |  |
| **Absent** | 41 | 53 | 30 | 11 |  |  |
| **Present** | 1 | 2 | 8 | 8 |  |  |
|  |  |  |  |  |  |  |
